# Urban-rural disparities in immunization coverage among children aged 12–23 months in Ethiopia: multivariate decomposition analysis

**DOI:** 10.1186/s12913-023-09940-4

**Published:** 2023-09-07

**Authors:** Melash Belachew Asresie, Gedefaw Abeje Fekadu, Gizachew Worku Dagnew

**Affiliations:** Department of Reproductive Health and Population Studies, School of Public Health, College of Medicine and Health Sciences, Bahir Dar University, Bahir Dar, Ethiopia

**Keywords:** Immunization, Disparities, Children aged 12–23 months, Decomposition, Ethiopia

## Abstract

**Background:**

Immunization is one of the most cost-effective public health interventions for improving children’s health and survival. In Ethiopia, low immunization coverage and disparity across residences are major public health problems. However, the factors that contributed to the urban-rural disparity have not been thoroughly investigated. Therefore, the objective of this study was to examine the change and contributing factors in full immunization coverage across geographic locations (urban-rural) in Ethiopia.

**Methods:**

We analyzed data on children aged 12 to 23 months obtained from the 2019 mini-Ethiopian demographic and health survey. A total of 996 weighted samples (299 in urban and 697 in rural areas) were included in the analysis. A multivariate decomposition analysis technique was used to determine the disparity and identify factors that contribute to the disparity across geographical locations. Statistical significance was defined at a 95% confidence interval with a p-value of less than 0.05.

**Results:**

The percentage of children aged 12–23 months who received full immunization increased from 36.84% (95% CI:31.59, 42.41) in rural areas to 64.59% (95% CI:47.10, 78.89) in urban areas. The decomposition analysis showed that the observed urban-rural disparity was attributed to a change in the effect of population characteristics (coefficient) across residences. Specifically, receiving 1–3 (β = 0.0895, 95% CI: 0.0241, 0.1550) and 4 or more (β = 0.1212, 95% CI: 0.0224, 0.2199) antenatal care visits, delivering at a health facility (β = 0.1350, 95% CI: 0.0227, 0.2472), and the source of information about immunization status from vaccination cards (β = 0.2666, 95% CI:0.1763, 0.3569) significantly contributed to the widening urban-rural disparity. On the other hand, being of high wealth status (β=-0.141, 95% CI: -0.1945, -0.0876), receiving postnatal care (β=-0.0697, 95% CI: -0.1344, -0.0051), and having four or more living children (β=-0.1774, 95% CI: -0.2971, -0.0577) significantly contributed to narrowing the urban-rural disparity.

**Conclusions:**

There was a significant urban-rural disparity in immunization coverage in Ethiopia, with urban children more likely to complete immunization. The change in the composition of population characteristics was not significant for the observed disparity. The observed disparity in full immunization coverage was mainly driven by the coefficients related to maternal healthcare utilization, household wealth status, the number of living children, and the source of immunization information. Therefore, strengthening maternal health services utilization, encouraging mothers to maintain their children’s immunization records, and addressing economic inequality, particularly in rural areas, may narrow the urban-rural disparity and enhance immunization coverage nationwide.

## Background

The global situation regarding children’s survival has shown improvement, yet it remains a significant public health concern. Approximately 6 million children died before reaching their fifth birthday worldwide in 2016, with Sub-Saharan Africa being responsible for half of these deaths [1]. Pneumonia, diarrhea, and malaria collectively account for over one-third of under-five mortalities globally and 40% of under-five mortalities in sub-Saharan Africa [1]. Immunization is one of the most effective public health tools for improving child health and preventing mortality from vaccine-preventable illnesses. Annually, it saves 2 to 3 million children from mortality [2, 3]. It has also been recognized as a tool in achieving the 2030 Sustainable Development Goal target of reducing under-five mortality to 25 per 1000 live births [4]. The World Health Organization launched the Expanded Program of Immunization (EPI) in 1974 as a public health initiative aimed at improving child health. The immunization coverage rate has been successfully increasing from 5% at the inception to 86% in 2019 [5]. Despite these efforts, an estimated 18.2 million infants didn’t receive the initial dose of the Diphtheria-Pertussis-Tetanus (DPT) vaccine in 2021, as well as 6.8 million children vaccinated partially in the same year [6]. Due to that, Pneumonia and diarrhea are still the major cause of under-five deaths [1]. About 60% of unvaccinated children live in 10 countries (Angola, Brazil, the Democratic Republic of the Congo, Ethiopia, India, Indonesia, Myanmar, Nigeria, Pakistan, and the Philippines [6].

Moreover, the disparity between urban and rural areas in immunization uptake is a public health concern in many countries. Numerous studies conducted in various parts of the world revealed that immunization coverage in rural areas is lower than in urban areas [7–9]. The age of the mother, birth order, wealth index, working status, distance to health facilities, media exposure, maternal health service utilization, and sex of the baby was identified as contributing factors to the urban-rural disparity in full immunization [9, 10].

Ethiopia launched the EPI program in 1980 and provides for free at the community and facilities levels [11]. Currently, one dose of Bacillus-Guerin (BCG), three doses of Oral Polio Vaccine (OPV), three pentavalent doses (Diphtheria, Tetanus, Pertussis, Hepatitis B, and Haemophilus influenza type B vaccine), three doses of the pneumococcal conjugate, two doses of rotavirus, and one dose of measles vaccine are recommended for children in their first year of life [12]. In addition, there has been a recent update in the vaccination schedule where the second dose of the measles vaccine is now administered at 13 months of age [12]. The Ethiopian government launched the health extension program in 2005 to promote child and maternal health and increase the utilization of health services [13]. EPI is one of the components of the health extension package offered routinely at health facilities and health posts, as well as through outreach [13]. There has been a remarkable improvement in child health in Ethiopia recently. Under-five mortality fell by 67% from 166 to 2000 to 55 deaths per 1,000 live birth in 2019 [12, 14]. Full immunization coverage in Ethiopia increased from 14% to 2000 to 43% in 2019 [12, 14], but is lagging behind the national target. Household and maternal sociodemographic characteristics and maternal health service use-related factors are the driving factors for the increased uptake [15, 16]. However, there is an urban-rural disparity in full immunization coverage, a higher proportion of urban children complete basic immunization compared to rural children [12, 14], despite the global public health emphasis on health equity and quality healthcare services [17, 18] and the inclusion of these priorities in the Ethiopian health sector transformation plan [19]. However, factors that contributed to the urban-rural disparity in immunization coverage have not been well explored. Exploring factors contributing to the disparity in immunization uptake has been critical in improving immunization coverage and minimizing inequality in service uptake across geographical locations (urban and rural). In addition, the findings of this study are useful to achieve sustainable development goal three, eliminating preventable deaths of newborns and children under the age of five (3.2), and achieving universal health coverage (3.8) [20].

Therefore, the objective of this study was to measure urban-rural disparity in immunization coverage and identify factors that contribute to the disparity using a multivariate decomposition analysis technique.

## Methods

### Data source

We used the 2019 mini–Ethiopian Demographic and Health Survey (EDHS) data, which is administrated across nine regions and two cities [12].

### Study design and period

The EDHS is a national, regional, and residential representative community-based cross-sectional survey. The 2019 mini-EDHS data were collected from March 21, 2019, through June 28, 2019 [12].

### Study population and sample size

The study population comprised children aged 12–23 months during the data collection period. The 2019 mini-EDHS gathered data on immunization coverage from a sample of weighted 1,026 children aged 12–23 months. After 30 individuals were removed due to missing observations in certain crucial variables, a total of 996 weighted samples were included in the final analysis (299 in urban and 697 in rural).

### Sampling technique and procedure

The EDHS used a two-stage stratified cluster sampling technique. The Ethiopia Population and Housing Census (PHC), conducted in 2007, served as the sampling frame for the EDHS. The census frame has a list of the enumeration areas (EAs) established for the 2007 PHC. It includes information about the EA’s location, types of residence, and the expected number of residential households in each region. Each region was initially classified into urban and rural areas, yielding 21 sampling strata. EAs were chosen in the first stage with a probability proportional to EA size based on Ethiopia’s 2007 PHC. For 2019 min-EDHS, 305 enumeration areas (93 in urban and 212 in rural areas) were chosen with probability proportionate to enumeration size. Then, from January to April 2019, a household listing operation was conducted in all of the enumeration areas that were chosen. In the second stage, using an equal probability systematic selection method, 30 households per cluster were chosen. Then, if any of the selected families had a child aged 12–23 months, information regarding the child’s vaccination status was gathered. The mothers were requested to present the Infant Immunization Card or health card used to document their child’s immunizations. If the vaccination information was not recorded on these cards, the mother was asked to recall whether the specific vaccination had been administered to the child. In cases where the mother could not provide the Infant Immunization Card, she was asked to remember if the child had indeed received the recommended vaccines. If she confirmed the child’s vaccination, additional inquiries were made regarding the number of vaccine doses received for each vaccine [12].

### Measurements

The outcome variable for this study was immunization status, which was classified dichotomously as “Yes/No”. A child who received one dose of Bacillus Caliette-Guerin (BCG), three doses of combined Diphtheria-Pertussis-Tetanus (DPT), three doses of polio (excluding polio zero), and one dose of measle was considered as fully immunized or coded as “Yes (1)” and “No (0)” if the child had no received or missed any of the basic vaccines mentioned above. Each vaccine antigen variable was classified as “1” for a child who received the vaccine dose and “0” for those who didn’t receive it. These values were then added together to calculate the immunization status. If a child received all of the recommended doses of the above-mentioned vaccines, the immunization status was re-coded as “1” (full immunization) and “0” (one or incomplete immunization) if at least one of the vaccine doses was missing or if a child had not received any vaccine” [12, 21].

#### Grouping variable

Geographic location or place of residence was used to categorize study participants (urban and rural).

#### Independent variables

After reviewing different literature and the availability of the variables in the 2019 mini-EDHS, the following variables were selected. Sociodemographic factors such as mothers’ age at the time of the interview were categorized as an ordinal variable as 15–24, 25–34, and 35–49 years, and the mother’s educational status was measured as an ordinal categorical variable (no education, primary, and secondary or above), religion as measured as a nominal categorical variable (Christian, and other (Muslim, tradition, and others)), marital status was measured as a nominal categorical variable (single and married), household members were categorized as ≤ 5, and ≥ 6), exposure to media was measured as a nominal categorical variable ( no and yes), number of under-five children in the household was categorized as ≤ 1 and ≥ 2, number of living children was measured as ≤ 3 and ≥ 4, sex of children (female and male), birth order was measured as an ordinal categorical variable (1, 2–3, and 4 or above), and source of immunization information was measured as a nominal categorical variable as mother self-report and cards. Enabling factors: Place of birth was measured as a nominal categorical variable (home, and health facility), antenatal care (ANC) visits were measured as an ordinal categorical variable (no, 1–3 visits, and 4 or more visits), postnatal care (PNC) visit was measured as a nominal categorical variable (no and yes).

#### Wealth index

The poor wealth index category was created by merging poorer and poorest and the variable rich was constructed by merging richer and richest because some categories were small for statistical analysis. A woman was considered exposed to media if she read newspapers or magazines or watched television or listened to the radio at least once a week. These variables were measured based on the mother’s reports.

### Data management and analysis

The samples were weighted to correct disproportionate sampling and non-response using STATA software version 15.1. The “svyset” command was also applied to handle the effect of complex sample surveys in the EDHS.

A multivariate decomposition non-linear model of analysis was performed using the “mvdcmp” command to see the disparity in full immunization coverage between rural and urban and identify factors that contributed to the disparity across the residences. Multivariate decomposition analysis is used to detect the changes and identify the source of the changes between the two groups (for this study rural-urban) [22]. This provided detailed information about the source of the change between the two groups due to changes in the composition of explanatory variables (endowment) or the effect of explanatory variables (coefficient), as well as the contribution of specific characteristics to the changes. In this study, we did the decomposition analyses across the residences.

#### Full-immunization coverage across residences

The purpose of the multivariate decomposition analysis was to understand the disparity in immunization coverage and identify factors that contributed to the disparity by residences. For this analysis, rural was coded as “0” and urban as “1”.

The difference in both population composition and the effect of the population characteristics between urban and rural were considered to understand the sources of factors contributing to the disparity across residences. Logit-based decomposition analysis technique was used for the analysis of factors contributing to the disparity in immunization coverage to identify the sources of disparity between urban and rural areas. The disparity in immunization coverage between urban and rural can be attributed to the difference in composition in characteristics across the residences and the effect of those variables. Hence, the observed difference in full immunization coverage between the rural and urban was decomposed into characteristics (E) and Coefficients (C).

The logit-based difference was decomposed as

1 $$Y = F(\frac{{{e^x}^\beta }}{{1 + {e^x}^\beta }})$$

2 $$Ya - Yb = F(\frac{{{e^{xa\beta a}}}}{{1 + {e^{xa\beta a}}}}) - F(\frac{{{e^{xb\beta b}}}}{{1 + {e^{xb\beta b}}}})$$

3 $$Y = [F(\frac{{{e^{xa\beta a}}}}{{1 + {e^{xa\beta a}}}}) - F(\frac{{{e^{xb\beta b}}}}{{1 + {e^{xb\beta b}}}})] + [F(\frac{{{e^{xa\beta a}}}}{{1 + {e^{xa\beta a}}}}) - F(\frac{{{e^{xb\beta b}}}}{{1 + {e^{xb\beta b}}}})]$$

where: Y is the dependent variable, X is the independent variable, β is the coefficient, and F is a differential logistic function of X and Y.

Before multivariate decomposition analysis, multi-collinearity between each independent variable was checked. Statistical significance was determined at a p-value of less than 0.05.

## Results

### Characteristics of children and mothers

About 49% and 52% of rural and urban children were from mothers aged 25–35 years respectively. 9% of rural children and 29% of urban children were born to mothers who attended secondary or above education. About 22.4% of rural children were born in rich households, as compared to 82% of urban children. The percentage of children whose mothers received four or more ANC visits during the index babies’ pregnancy was 37.6% in rural and 59% in urban areas. About 46% of rural and 74.4% of urban children were delivered at facilities. As the chi-square test result showed that, there was a statistically significant difference between urban and rural areas in educational status, exposure to media, ANC visit, place of birth, PNC visit, birth order, number of living children, and source of information about immunization status at p-value < 0.05 (Table 1).

**Table 1:** Sociodemographic characteristics of the participants in Ethiopia, Mini-EDHS 2019

Variables		Frequency (%)		Chi-square (p-value)
		Rural (N = 697)	Urban (N = 299)	
Mother’s current age in years				
	15–24	31.8	28.8	1.33(0.810)
	25–34	48.7	52.2	
	35–49	19.5	19.0	
Mother’s educational status				
	No education	52.8	27.4	98.26(< 0.001)
	Primary	39.4	(43.8	
	Secondary or above	7.8	28.7	
Mother’s religion				
	Christian	62.7	67.6	2.42(0.638)
	Other	37.3	32.6	
Household wealth index				
	Poor	54.7	13.5	345.77(< 0.001)
	Middle	22.9	4.5	
	Rich	22.4	82.0	
Number of household members				
	≤ 5	52.6	63.8	12.63(0.089)
	≥ 6	47.4	36.2	
Exposure to media				
	No	73.5	34.3	149.92(< 0.001)
	Yes	26.5	65.7	
ANC Visits				
	No visits	31.2	11.4	67.98 (0.001)
	1–3 visits	31.2	29.6	
	4 or above	37.6	59.0	
Place of Delivery				
	Home	53.4	25.6	78.78(0.013)
	Health facilities	46.6	74.4	
PNC visits				
	No	70.0	46.1	56.06(0.001)
	Yes	30.0	53.9	
Birth order				
	1	20.6	28.9	33.56(0.028)
	2–3	34.5	44.0	
	≥ 4	44.9	27.1	
Sex of baby				
	Male	48.9	49.0	0.01(0.982)
	Female	51.1	51.0	
Number of under 5 children in the household				
	≤ 1	42.9	43.7	0.06(0.901)
	≥ 2	57.1	56.3	
Number of living children				
	≤ 3	58.0	72.9	23.82 (0.0.30)
	≥ 4	42.0	27.1	
Source of information about immunization				
	Mother	66.4	40.0	68.15(0.002)
	Card	33.7	60.0	

### Urban-rural disparity in immunization coverage

**Fig. 1:**
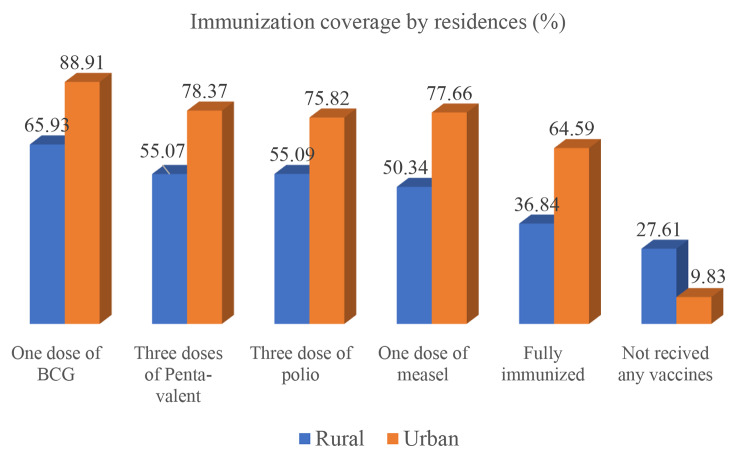
Childhood immunization coverage by residential in Ethiopia, mini-EDHS 2019

Full immunization coverage increased from 36.84% (95% CI: 31.59–42.41) in rural areas to 64.59% (95% CI: 47.10-78.89) in urban areas. Besides, about 28% (95% CI: 22.37–33.54) of rural children and 10% (95% CI: 4.67–19.52) of urban children have not received any vaccines (Fig. 1).

### Residential immunization coverage by participants’ characteristics

Full immunization coverage varied by women’s characteristics in the two settings. Nearly half (49%) of rural and more than three fourth (79%) of urban children born to mothers aged 35 to 49 years were fully immunized, as compared to 34% of rural and 55% of urban children born to mothers aged 15 to 24 years. Approximately 46% of rural children and 76% of urban children born to mothers with secondary or higher education were fully immunized, compared to 31% and 48% of urban and rural children who were born to mothers with no formal education, respectively. The chi-square test result revealed that in rural children, full immunization coverage was associated with the mother’s educational status, household wealth index, number of household members, ANC visits, place of delivery, PNC visit, number of under-five children in the household, and source of information about children’s immunization status at p-value < 0.5, whereas in urban children, the only source of information about children’s immunization was associated with full immunization (Table 2).

**Table 2:** Residential distribution of full immunization coverage among children 12–23 months in Ethiopia, Mini EDHS 2019

Variables		Full immunization coverage by residential (%)				Point difference (urban-rural)
		Rural (N = 697)	Chi-square (p-value)	Urban (N = 299)	Chi-square (p-value)	
Mother’s current age in years						
	15–24	34.3	3.2 (0.442)	54.9	7.8 (0.289)	20.6
	25–34	36.0		64.5		28.5
	35–49	43.2		79.4		36.2
Mother’s educational status						
	No education	30.9	12.1 (0.064)	48.1	12.8 (0.246)	17.2
	Primary	43.0		67.7		24.7
	Secondary or above	45.8		75.5		29.7
Mother’s religion						
	Christian	39.9	4.9 (0.271)	64.7	0.1 (0.986)	24.8
	Other	31.6		64.6		33.0
Household wealth index						
	Poor	29.2	37.0 (0.002)	87.5	10.5 (0.207)	58.3
	Middle	35.7		45.7		10.0
	Rich	56.7		61.9		5.2
Number of household members						
	≤ 5	42.2	9.7 (0.025)	63.6	0.2 (0.841)	21.4
	≥ 6	30.9		66.4		35.5
Exposure to media						
	No	34.5	4.8 (0.165)	66.9	0.3 (0.826)	32.4
	Yes	43.4		63.7		20.3
ANC Visits						
	No visits	20.9	52.9 (< 0.001)	36.0	16.7 (0.067)	15.1
	1–3 visits	34.0		58.4		24.4
	4 or above	52.4		73.2		20.8
Place of Delivery						
	Home	25.0	49.3 (< 0.001)	40.9	21.9 (0.171)	15.9
	Health facilities	50.0		72.7		22.7
PNC visits						
	No	27.0	70.3 (< 0.001)	56.0	7.2 (0.301)	29.0
	Yes	60.0		72.0		12.0
Birth order						
	1	39.4	2.5 (0.517)	60.0	3.7 (0.611)	20.6
	2–3	39.4		71.0		31.6
	≥ 4	33.7		59.1		25.4
Sex of baby						
	Male	37.0	0.01 (0.941)	66.4	0.4 (0.652)	29.4
	Female	36.6		62.9		26.3
Number of under 5 children in the household						
	≤ 1	43.7	10.9 (0.025)	68.0	1.0 (0.652)	24.3
	≥ 2	31.7		61.9		30.2
Number of living children						
	≤ 3	39.3	2.6 (0.209)	66.6	1.3 (0.628)	27.3
	≥ 4	33.4		59.1		25.7
Source of information about immunization						
	Mother	30.5	24.4 (0.007)	34.2	69.6 (< 0.001)	3.7
	Card	49.4		84.8		35.4

### Factors contributing to the urban-rural disparity in full immunization coverage

There was a 27.75% (95%CI:21.16–34.34) disparity in full immunization coverage between urban and rural areas, with children in urban more likely to complete immunization. The multivariate decomposition analysis showed that the disparity in immunization coverage across residences was significantly attributed to the change in the effect of population characteristics (coefficient) only. It attributed 96.06% of the explained disparity (β = 0.2666,95%CI: 0.1763, 0.3569). If the behavior of the population in urban and rural areas had been similar, the urban-rural disparity in full immunization coverage would have been reduced by 96.06%. The change in the effect of characteristics (coefficient) among mothers who had ANC and PNC visits, were delivered at a health facility, were from rich households, had four or more living children, and had a vaccination card as a source of information about childhood vaccination status significantly contributed to the urban-rural disparity in full immunization coverage. If the coefficient of women from rich households’ and who had four or more living children were identical across residences, the disparity in full immunization coverage would have increased by 50.81% (β=-0.141 -0.1945, -0.0876) and 63.93% (β=-0.1774, 95% CI-0.2971, -0.0577), respectively. Whereas, if the coefficient of women who received 1–3 and 4 or more ANC visits had remained identical in urban and rural areas, the disparity in full immunization coverage would have decreased by 32.26% (β = 0.0895, 95% CI: 0.0241, 0.1550) and 43.66% (β = 0.1212, 95% CI: 0.0224, 0.2199), respectively. Similarly, if the coefficient of women who delivered at a health facility was similar across residences, the disparity in full immunization coverage would have decreased by 48.64% (β = 0.1350, 95% CI: 0.0227, 0.2472). The disparity in full immunization coverage across the residences would have decreased by 58.76% (β = 0.2666, 95%CI:0.1763, 0.3569) if the coefficient of women who kept a vaccination card was identical between urban and rural areas. On the other hand, if the coefficient of women who received PNC visits were identical in urban and rural areas, the disparity in full immunization coverage would have increased by 25.14% (β=-0.0697, 95%CI: -0.1344, -0.0051).

The difference in composition of population characteristics (β = 0.0109, 95%CI: -0.0581, 0.0799) was attributed to 3.96% of the explained disparity but was not statistically significant. A positive coefficient would contribute to the urban-rural disparity in immunization coverage, and it is interpreted as supporting (increasing) the disparity, which is in favor of urban children. A negative coefficient would contribute to the urban-rural full immunization coverage disparity, and it is interpreted as working to reduce the disparity, which is in favor of rural children (Table 3).

**Table 3:** Residential decomposition disparity in full immunization coverage among children aged 12–23 months in Ethiopia, Mini-EDHS 2019

		Differences due to characteristics (E)		Difference due to coefficient (C)		Total
		Coefficient	Percent	Coefficient	Percent	
Mother’s current age in years						
	15–24	1				
	25–34	0.0003 (-0.0024, 0.0030)	0.11	-0.0335 (-0.1317, 0.0648)	-12.06	-11.95
	35–49	0.0006 (-0.0046, 0.0058)	0.22	0.0607 (-0.0084, 0.1297)	21.87	22.09
Mother’s educational status						
	No education	1				
	Primary	-0.0007 (-0.0.0078, 0.0062)	-0.26	0.0095 (-0.1022, 0.1212)	3.42	3.16
	Secondary or above	-0.0023 (-0.0309, 0.0264)	-0.81	0.0082 (-0.0229, 0.0392)	2.94	2.13
Mother’s religion						
	Christian	1				
	Other	-0.0016 (-0.0148, 0.0117)	-0.57	-0.0434 (-0.1003, 0.0127)	-15.79	-16.36
Household wealth index						
	Poor	1				
	Middle	-0.0062 (-0.0550, 0.0426)	-2.58	-0.0283 (-0.1094, 0.0529)	-10.18	-12.76
	Rich	0.0910 (-0.6523, 0.8345)	29.19	-0.1410 (-0.1945, -0.0876) ***	-50.81	-21.62
Household members						
	≤ 5	1				
	≥ 6	-0.0004 (-0.0066, 0.0057)	-0.15	0.0352 (-0.0431, 0.1135)	12.68	12.53
Exposure to media						
	No	1				
	Yes	0.0246 (-0.1869, 0.2361)	8.86	-0.0506 (-0.1133, 0.0120)	-18.24	-9.38
ANC Visits						
	No visits	1				
	1–3 visits	0.0014 (-0.0108, 0.0136)	0.52	0.0895 (0.0241, 0.1550) **	32.26	32.78
	4 or above	-0.0254 (-0.2400, 0.1891)	-9.16	0.1212 (0.0224, 0.2199) *	43.66	34.5
Place of delivery						
	Home	1				
	Health facilities	-0.0237 (-0.2302, 0.1829)	-8.53	0.1350 (0.0227, 0.2472) *	48.64	40.11
PNC visits						
	No	1				
	Yes	0.0091 (-0.0724, 0.0906)	3.29	-0.0697 (-0.1344, -0.0051) *	-25.14	-21.85
Sex of baby						
	Male	1				
	Female	0.0001 (-0.0002, 0.0002)	-0.01	-0.0250 (-0.1073, 0.0573)	-9.01	-9.02
Number of under 5 children in the household						
	≤ 1	1				
	≥ 2	0.0002 (-0.0002, 0.0002)	0.07	0.0641 (-0.0514, 0.1796)	23.1	23.17
Number of living children						
	≤ 3	1				
	≥ 4	-0.0170 (-0.1613, 0.1273)	-6.13	-0.1774 (-0.2971, -0.0577) **	-63.93	-70.06
Source of information about immunization						
	Mother	1				
	Card	-0.0392 (-0.3736, 0.2953)	-14.11	0.1631 (0.1189, 0.2073) ***	58.76	44.65
Total		0.0109 (-0.0581, 0.0799)	3.94	0.2666 (0.1763, 0.3569) ***	96.06	100

Key: 1 reference, * significant at < 0.05, ** significant at < 0.01, and *** significant at < 0.001

## Discussion

The main objective of this study was to assess the disparity in full immunization coverage between rural and urban children and identify factors that contributed to the disparity using decomposition analysis. Understanding these factors contributed to the disparity across the residence is crucial for decision-makers and partners involved in children’s health programs, as it enables them to develop targeted interventions and strategies aimed at improving immunization coverage in Ethiopia and reducing the urban-rural gap.

The findings of this study revealed that there was a statistically significant difference in full immunization coverage between children in urban and rural areas. Full immunization coverage or complete immunization status was found to be 28% higher among children in urban areas than in rural areas. This finding was in line with previous studies conducted in low and middle-income countries [7–10, 16, 23]. The possible justification for lower full immunization coverage in rural areas compared to urban areas might be attributable to the difference in accessibility to the health facility across residences. Private and governmental health facilities in Ethiopia are concentrated in urban areas, allowing greater access to the healthcare system [24]. Conversely, in rural areas, the scarcity of health facilities and intermittent shortages of essential medicines are the main challenges [24, 25]. Contrary to this study, studies out of Ethiopia, found that rural children were more likely to be fully immunized [26–28]. The possible reason for this is the presence of substantial barriers to vaccination services in an urban setting with a large underserved population in slums and informal settings.

In this study, the disparity in full immunization coverage between urban and rural areas was significantly attributed only to the change in the effect of population characteristics (coefficient). The change in the effect of population characteristics (coefficient) across residences was attributed to 96.06% of the explained disparity. The change in the coefficient of women from rich households, women who received ANC and PNC visits, delivered at health facilities, had four or more living children, and kept immunization records as a source of information about immunization status, was significantly associated with the disparity in full immunization coverage across residences.

This study revealed that change in the coefficient of women from rich households had a notable effect in reducing the urban-rural disparity in full immunization coverage. This finding was supported by previous studies [9, 16]. The possible justification might be that, despite immunization being provided free of charge in Ethiopia, women residing in rural areas may face challenges in accessing healthcare facilities due to geographical distances. They may need to undertake long journeys to reach the facilities. In contrast, women from wealthier households may have the financial means to cover transportation costs and other associated fees. Additionally, wealthier individuals often have better access to mass media, which can provide them with information about immunization and healthcare services [29].

The findings of this study revealed that the change in the coefficient of women who had ANC and delivered at a health facility across residences was an important predictor widening the urban-rural disparity in full immunization coverage. This finding was supported by a previous study [16]. One potential explanation is that the presence of health facilities in urban areas allows women to have better access to maternal health services. This increased accessibility leads to higher utilization of these services among urban women. Additionally, regular interaction with healthcare professionals provides a valuable opportunity for mothers to receive information about childhood immunization. This, in turn, enhances mothers’ confidence and encourages them to utilize childhood immunization services [28, 30]. Moreover, infants born at health facilities also have the opportunity to receive the first dose of BCG after birth [16]. Furthermore, in urban areas, the majority of women are educated and have access to diverse healthcare information sources, which may augment their knowledge about childhood immunization alongside information received during ANC and delivery [31–34]. Nevertheless, the inadequate quality of ANC and institutional delivery services, along with the limited resources and the considerable distance to healthcare facilities in rural areas [25, 35], may not offer the same level of opportunities as those available in urban settings.

The change in the coefficient of women who had PNC visits across residences significantly contributed to the narrowing of the disparity in full immunization coverage. This could be due to the fact that rural resident women who had PNC visits are more aware of childhood immunization than women who had not received PNC services, since PNC services provide excellent opportunities to receive vaccine services and appropriate messages about childhood immunization [36]. Rural women visiting a health facility for maternal and child health services might be the only source to get information about immunization because of a lack of access to media exposure, primarily due to limited electricity infrastructure [37]. However, urban resident mothers may have obtained information about childhood immunization through various sources of media, even in cases where they did not receive PNC visits. Studies showed that mothers who had access to mass media were more inclined to ensure complete immunization for their children compared to mothers who had limited exposure to mass media [38, 39].

The finding of this study also showed that the change in the coefficient of mothers with four or more living children across the residences narrowing the urban-rural gap in full immunization coverage. This could be due to the knowledge and experience gained by women through their previous childbirths during their engagement with maternal and child health services [40]. First-time mothers may exhibit lower levels of confidence and anticipate the need for greater support [41]. On the contrary, higher birth order is a symbol of bigger families and the characteristics of poor families, which may worsen access to healthcare [42]. In poor families where the responsibility for their children’s care and household chores is left to mothers, they may not have time to visit a vaccination center.

In this study, we found that the change in the coefficient of women who were keeping children’s immunization cards/records across residences significantly contributed to the widening of the urban-rural disparity in full immunization coverage. This finding was supported by other previous studies [43, 44]. The explanation for this could be that the mothers who kept the immunization card reflect how much attention they paid to immunization or how much knowledge they had about it, which may trigger memories in the mothers and other family members when they see it. However, it may not work for rural women because the majority of them are uneducated and thus unable to recognize whether their children have been immunized or not [45].

In this study, the difference in composition of population characteristics was attributed to 3.96% of the explained disparity but was not significantly associated with it. The reason for this might be a small difference in the composition of the explanatory variables across residences. The chi-square test result in this study revealed that the age of mothers, household wealth index, birth order, sex of the baby, and the number of under-five children in the households were not significantly different in composition between urban and rural areas.

### Limitations of the study

Although this study revealed the urban-rural disparity in full immunization coverage and identified the factors that contributed to the disparity, some important vaccination-related variables are missing from this analysis because they were not collected at the primary source.

## Conclusions

The objective of this study was to investigate urban-rural disparity in immunization coverage. The findings revealed that there was a statistically significant disparity in full immunization coverage across residences in Ethiopia, with children in urban areas more likely to complete immunization. The decomposition analysis showed that only the change in the effect of population characteristics (coefficient) was significantly associated with the disparity. Particularly, the change in the coefficient of women who were from rich households, who had received ANC and PNC visits, delivered at health facilities, who had four or more living children, and who kept immunization records as a source of information about immunization status was significantly associated with the disparity in full immunization coverage across residences. This emphasizes the significance of increasing efforts to improve accessibility and utilization of maternal health services, along with promoting awareness about child immunization in rural areas. Moreover, addressing economic disparities and encouraging mothers to maintain their children’s immunization records are vital steps to be taken. Additionally, individuals and organizations working towards equitable immunization coverage for all children should actively tackle the challenges posed by households with four or more living children. Furthermore, this study highlights the importance of conducting further research on the equitable distribution of vaccine supplies and program monitoring.

## Data Availability

The authors used the 2019 EDHS data for this analysis. Since the dataset is publicly available, interested researchers can access it by using the link: https://dhsprogram.com/data.

## List of abbreviations

ANC: Ante Natal Care
EDHS: Ethiopian Demographic and Health Survey
PNC: Post Natal Care
